# A Clustered Case Series of Mucorales Detection in Respiratory Samples from COVID-19 Patients in Intensive Care, France, August to September 2021

**DOI:** 10.3390/jof8030258

**Published:** 2022-03-03

**Authors:** Emilie Guemas, Sophie Cassaing, Sandra Malavaud, Judith Fillaux, Pamela Chauvin, Lucie Lelièvre, Stéphanie Ruiz, Béatrice Riu, Antoine Berry, Xavier Iriart

**Affiliations:** 1 Department of Parasitology and Mycology, Toulouse University Hospital, 31059 Toulouse, France; guemas.e@chu-toulouse.fr (E.G.); cassaing.s@chu-toulouse.fr (S.C.); fillaux.j@chu-toulouse.fr (J.F.); chauvin.p@chu-toulouse.fr (P.C.); berry.a@chu-toulouse.fr (A.B.); 2 Institut Toulousain des Maladies Infectieuses et Inflammatoires (Infinity), Université Toulouse, CNRS UMR5051, INSERM UMR1291, UPS, 31024 Toulouse, France; 3 RESTORE (Geroscience and Rejuvenation Research Center), UMR 1301-Inserm 5070-CNRS EFS, Université Paul Sabatier, 31100 Toulouse, France; 4 Healthcare Associated Risk Prevention Unit (UPRIAS), Toulouse University Hospital, 31059 Toulouse, France; malavaud.s@chu-toulouse.fr; 5 Department of Infectious and Tropical Diseases, Toulouse University Hospital, 31059 Toulouse, France; lelievre.l@chu-toulouse.fr; 6 Department of Anesthesiology and Intensive Care, Toulouse University Hospital, 31059 Toulouse, France; ruiz.s@chu-toulouse.fr (S.R.); riu.b@chu-toulouse.fr (B.R.)

**Keywords:** fungal infection, COVID-19, mucormycosis, aspergillosis, *Aspergillus*, Mucorales, SARS-CoV-2, diagnosis, PCR, construction work

## Abstract

While COVID-19-associated pulmonary aspergillosis is now well described in developed countries, COVID-19-associated mucormycosis (CAM) has seemed to remain quite rare in Europe. A retrospective study was performed between March 2020 to September 2021 among COVID-19 adult patients in the intensive care unit (ICU) at Toulouse Hospital (Southern France). PCR screening on respiratory samples, which target *Aspergillus* or Mucorales DNA, were performed, and the number of fungal detections was evaluated monthly during the study period. During the 19 months of the study, 44 (20.3%) COVID-19 ICU patients had a positive PCR for *Aspergillus*, an overall rate in keeping with the incidence of ICU COVID-19 patients. Ten patients (7.1%) had a positive Mucorales PCR over the same period. Surprisingly, 9/10 had a positive *Mucor*/*Rhizopus* PCR in August-September 2021, during the fourth Delta SARS-CoV-2 variant wave. Epidemic investigations have identified a probable environmental cause linked to construction works in the vicinity of the ICU (high levels of airborne spores due to the mistaken interruption of preventive humidification and summer temperature). Even if CAM are apparently rare in Europe, a cluster can also develop in industrialised countries when environmental conditions (especially during construction work) are associated with a high number of COVID-19 patients in the ICU.

## 1. Introduction

Reports of secondary fungal infections following severe acute respiratory syndrome coronavirus 2 (SARS-CoV-2) have been increasing worldwide. COVID-19-associated pulmonary aspergillosis (CAPA) has been described since the beginning of the pandemic [1], especially in patients in the intensive care unit (ICU). CAPA, associated with poor outcomes, is now well described in Europe [2].

The recent increase in COVID-19 cases in India have been associated with increasing reports of COVID-19-associated mucormycosis (CAM) [3], and in this country is now a notifiable disease [4]. Nevertheless, CAM seemed to remain quite rare in Europe, at approximately 1% according to the French multicentre MYCOVID study [5].

Invasive mould infection (IMI) is challenging to diagnose in ICU patients with COVID-19. Molecular methods such as PCRs, which target *Aspergillus* or Mucorales DNA, are rapid sensitive diagnostic methods that also serve as a useful marker for monitoring the incidence of IMI in COVID-19 patients in the ICU. To date, there are no recommendations for the diagnosis and classification of CAM.

Here, we describe our experience of the longitudinal monitoring of COVID-19 patients hospitalised in the ICU with an increase in the detection of Mucorales in respiratory samples during the fourth COVID-19 wave in summer 2021 in France.

## 2. Materials and Methods

### 2.1. Study Design and Patients

We performed a retrospective study between 1 March 2020 to 30 September 2021 among COVID-19 adult patients with PCR-positive SARS-CoV-2 and hospitalised in ICU at Toulouse Hospital (Southern France). In Toulouse, the ICU is located at two sites, 6.8 km apart (the Rangueil and Purpan Hospitals). In accordance with French public health law [6], this protocol did not require approval from an ethics committee and was exempt from formal informed consent.

The number of COVID-19 patients hospitalised in ICU and the number of patients positive or negative for *Aspergillus* or Mucorales during the study period, were evaluated monthly using a review of the patients’ medical charts. The dates used for individual patients were the date of the first positive SARS-CoV-2 PCR and the date of the first positive fungal PCR. If all fungal PCR were negative, the date used was the date of the first test. For patients that are PCR-positive for Mucorales, data regarding patients’ demographics (biological sex, age), medical history (diabetes), ICU hospitalisation, antiviral therapeutic and biological data (variant SARS-CoV-2, fungal biomarkers in respiratory specimens and serum) were recorded. Antiviral therapeutics (dexamethasone and tocilizumab) were introduced according to the recommendations from the RECOVERY studies [7,8].

### 2.2. Fungal PCR and Culture

PCR screening for IMI among SARS-CoV-2 PCR-positive patients were performed with routine PCR testing of respiratory samples for *Aspergillus* and Mucorales, according to previous published protocols [9,10]. PCR tests for Mucorales detected the genera *Rhizomucor*, *Lichtheimia* and the undifferentiated pair *Mucor*/*Rhizopus*. The screening frequency depended on the clinicians and local ICU protocols. In clinical practice, fungal PCRs were mainly performed in severe patients with a long stay in intensive care.

Cultures were performed on respiratory samples using a Sabouraud agar medium. *Aspergillus* species were identified by morphology and/or matrix-assisted laser desorption ionization–time of flight mass spectrometry (MALDI-TOF MS) [11].

### 2.3. Statistical Analyses

Data were analysed with SIGMA Stat1 (2.03; Jandel Corporation, San Jose, CA, USA) software. Bivariate analysis was performed using the chi-squared test to compare the proportion of *Aspergillus*-positive patients or the *Aspergillus* species proportions according to the period. For continuous parameters, the data distribution was found to be non-Gaussian and values were reported as the median and interquartile range (IQR) [25%; 75%]. The differences were considered significant when *p* < 0.05.

## 3. Results

### 3.1. Patients Characteristics

**Figure 1:**
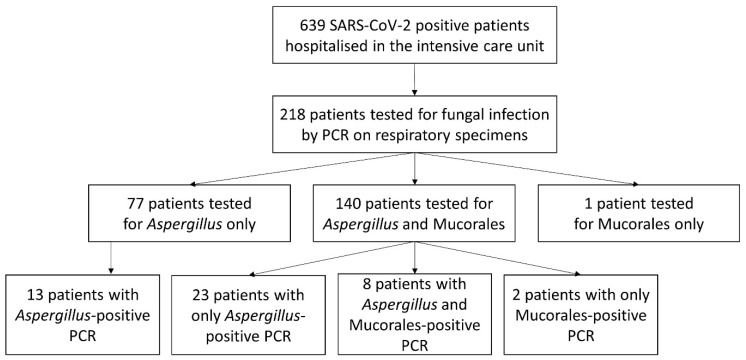
Flow chart of patients.

From 1 March 2020 to 30 September 2021, 639 PCR-positive SARS-CoV-2 patients were hospitalised in the ICU at Toulouse University Hospital (Figure 1). The male/female sex ratio was 2.1:1 and the median age was 63 [53; 73] years. Three hundred and eleven and 328 patients were hospitalised at the Rangueil and Purpan sites, respectively. At least one respiratory sample was taken from 218 of these patients for IMI-testing with PCR. One hundred and forty patients were tested for *Aspergillus* and Mucorales, 77 only for *Aspergillus* and 1 only for Mucorales.

### 3.2. Evaluation of the Incidence of Aspergillus- and Mucorales-Positive PCRs among ICU COVID-19 Patients

**Figure 2:**
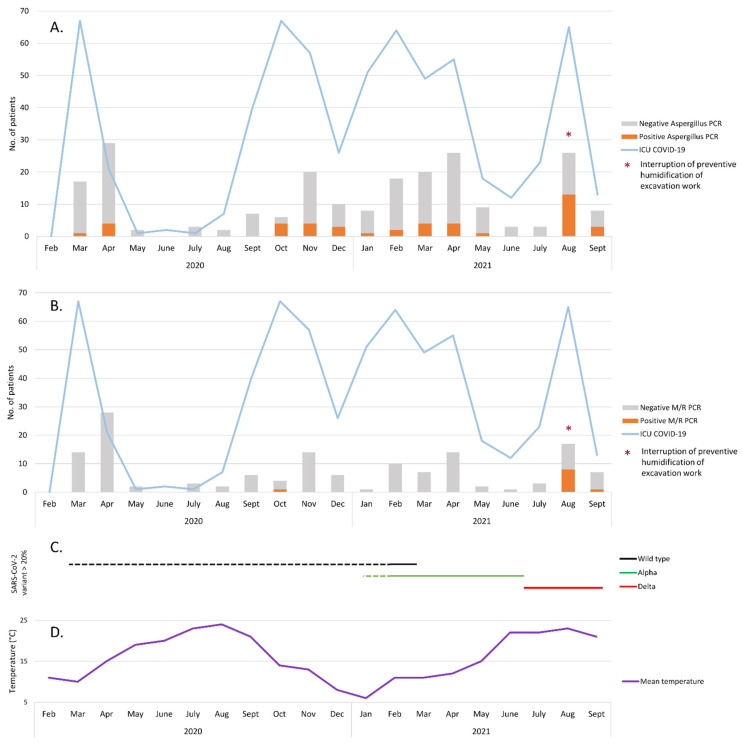
Number of COVID-19 patients hospitalised in ICU during the study period and results of respiratory sample screening for *Aspergillus* (**A**) or Mucorales (**B**). The dates used for individual patients were the date of the first positive SARS-CoV-2 PCR and the date of the first positive fungal PCR. If all fungal PCR were negative, the date used was the date of the first test. (**C**) SARS-CoV-2 variant circulation during the study period. Changes in the majority variants are represented by lines. Dotted lines are estimates. (**D**) Mean temperature (°C) in Toulouse during the study period [12].

Among the 217 patients who were PCR-tested for *Aspergillus*, 173 remained negative during their ICU stay and 44 (20.3%) had at least one positive PCR. One to 10 respiratory samples per patient were prescribed (median: 2). The overall rate of positive PCRs for *Aspergillus* was in keeping with the incidence of COVID-19 patients hospitalised in the ICU (the first four waves) (Figure 2A). Of the 141 patients whose respiratory samples were PCR-tested for Mucorales, 131 remained negative during hospitalisation in the ICU during the study period. Ten patients (7.1%) had at least one positive *Mucor*/*Rhizopus* PCR (Figure 2B). One to nine respiratory samples were prescribed per patient (median: 2).

### 3.3. Mucorales Infections among COVID-19-Patients in the ICU

Among the 10 COVID-19 patients whose respiratory sample tested PCR-positive for Mucorales (Table 1), the male/female sex ratio was 4:1 and the median age was 69 [58; 73] years. PCR-testing for Mucorales was positive at a median rate of 4.5 [4; 11] days after admission to the ICU. Diabetes, a common risk factor for mucormycosis, was reported for 20% (2/10) of the patients. Most of the patients were treated for COVID-19 infection with dexamethasone (9/10; 90%) and tocilizumab (7/10; 70%). Among the 10 patients, 90% (9/10) received antifungal drugs against Mucorales: 80% (8/10) received liposomal amphotericin B, 50% (5/10) isavuconazole and 20% (2/10) posaconazole. Five patients received two or three antifungal drugs against Mucorales. Mucormycosis was probable for at least three patients because *Mucor*/*Rhizopus* DNA was detected in the serum of these patients as well (mortality: 2/3; 66%). A concomitant *Aspergillus*-positive PCR was also observed in 80% (8/10) of the Mucorales-positive patients. The overall death rate reached 50% (5/10) in this cohort.

Surprisingly, the incidence of Mucorales-positive cases was very different in the successive waves of the COVID-19 pandemic (Figure 2B) and according to the SARS-CoV-2 variant prevalence (Figure 2C). During the first 17 months (March 2020 to July 2021), only one patient had a positive PCR (0.9%; 1/117), while 9/24 patients (patients B–J) (37.5%) had a positive *Mucor*/*Rhizopus* PCR in August–September 2021 (fourth wave). Interestingly, among patients B–J, 100% of those tested were positive for the Delta SARS-CoV-2 variant (7/7) and 77.8% (7/9) had a dexamethasone/tocilizumab combination therapy.

During the same period (August–September 2021), 47% (16/34) of the patients who were PCR-tested for *Aspergillus* had at least one positive result (Figure 2A), which is a high level compared to the previous waves (28/183; 15.3%, *p* < 0.001 chi-squared test). Moreover, fungal cultures in COVID-19 patients with an *Aspergillus*-positive PCR showed a higher proportion of non-*fumigati* species in August–September 2021 compared to the other 17 months of the study period (61.1% versus 30.4%, *p* = 0.049 chi-squared test).

**Figure 3:**
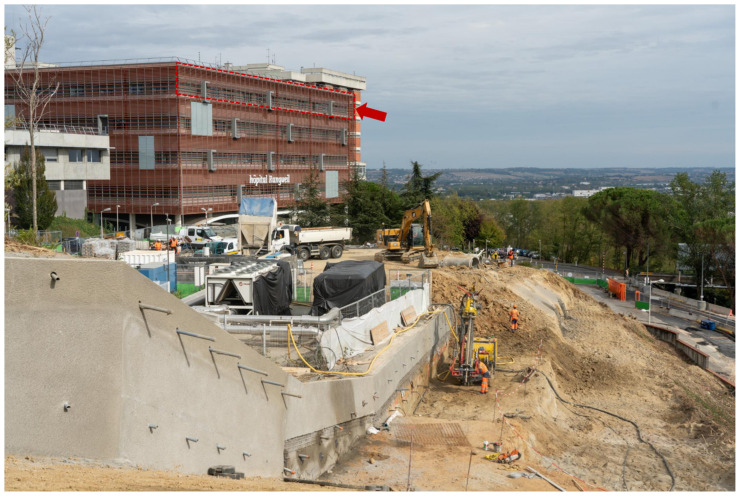
Picture showing the construction work and the localisation of the Rangueil ICU. The red box indicates the ICU floor where the COVID-19 patients were hospitalised. Photo courtesy of Tisséo Ingénierie—Airimage (© Tisséo Ingénierie—Airimage).

This alarmingly high number of positive fungal PCRs during the August–September period led us to investigate this “fungal epidemic”. The nine patients (patients B–J) (Table 1) were hospitalised on both ICU sites (six and three at Rangueil and Purpan, respectively). Among non-COVID-19 patients hospitalised in both ICU sites during the same study period (1 March 2020 to 30 September 2021), 94 and 51 patients were tested by PCR for *Aspergillus* and Mucorales, respectively. Among them, 15 and four had at least one positive PCR in respiratory samples, evenly distributed over the study period. In August 2021, construction and renovation works were taking place on both sites within 200 m of the ICU (Figure 3) in a context of summer weather with a mean temperature in Toulouse of about 23 °C (Figure 2D). Environmental tests (air and surface sampling) at the Rangueil ICU were positive for numerous filamentous fungi including *Rhizopus oryzae* in 6/38 samples (16%) and *Aspergillus* sp. in 6/38 samples (16%). At the time of the outbreak, preventive humidification of excavation work in the vicinity of the Rangueil ICU (<10 m) had been mistakenly interrupted. The Infection Prevention and Control team successfully implemented the following corrective measures: (i) resumption of sprinkling of the excavation, (ii) replacement of the ICU air handling unit inlet filters, (iii) countermanding the institutional COVID-driven recommendation to open the windows of tertiary rooms that were ill-adapted to the ICU controlled environmental zone, (iv) inactivation of the low pressure setting of COVID patients’ cubicles, thereafter maintained in isopression; and (v) intensification of bio-cleaning in cubicles and tertiary areas and storage rooms. During the month of September 2021, in parallel with the decline in the incidence of COVID-19 infections in the ICU, the level of Mucorales and *Aspergillus* detection drastically decreased and no other such episode has occurred since.

## 4. Discussion

In this paper, we report an alarming increase in *Mucor*/*Rhizopus*-positive PCRs in respiratory samples of COVID-19 patients hospitalised in the Toulouse university hospital ICU during the fourth wave in the summer of 2021.

Patients with severe COVID-19 pneumonia have emerged as a population with a high risk of fungal infections. The French multicentre MYCOVID study showed a high prevalence of CAPA (approximately 15%) and high mortality related to this infection in mechanically ventilated SARS-CoV-2 patients [5]. Moreover, during the spread of the SARS-CoV-2 Delta variant, numerous cases of mucormycosis in COVID-19 patients were reported worldwide, particularly in India [3]. In Europe, invasive mucormycosis seems to be less frequent (1% in the MYCOVID study) but isolated cases are occasionally observed [5,13,14]. A nationwide French publication has recently reported 17 cases of COVID-19 associated mucormycosis [15] which showed some differences with Indian cases, as with our study. The site of involvement was pulmonary for all the patients in our centre, as in almost all cases in the Danion et al. French study [15]. In India, the mucormycosis was essentially rhino-orbito-cerebral infections [3] but pulmonary forms of mucormycosis may have been underdiagnosed. Moreover, diabetes mellitus was a highly prevalent comorbidity factor in India [3], whereas it accounted for only 20% in our case series. Despite a similar mortality rate, the management of the patients differed significantly between Indian cases and patients of this study (more frequent use of tocilizumab and admission to ICU in this case series) [3].

Unlike the diagnosis of CAPA, for which recommendations have been published [2], to date no consensus criteria have been published for diagnosis of CAM. Cases were classified by some authors according to European Organization for Research and Treatment of Cancer and the Mycoses Study Group Education and Research Consortium (EORTC/MSGERC) criteria, with some modifications [15]. According to criteria proposed by Danion et al. [15], among the 10 COVID-19 patients whose respiratory sample tested PCR-positive for Mucorales, all of them had a host factor (diabetes mellitus and/or dexamethasone prescribed for COVID-19). Imaging-based criteria are difficult to use in COVID-19 patients in the ICU. On the one hand, they could have many atypical radiological features, and on the other hand, lesions suggestive of fungal infection can be hidden by lung involvement [2]. Although classification of Mucorales-positive patients is difficult, we can estimate that at least three patients in our case series can be classified as “probable mucormycosis” because *Mucor/Rhizopus* DNA was detected in the serum of these patients as well.

The sudden significant increase in Mucorales detection in our hospital in August 2021 led us to propose various hypotheses. First, an effect related to the SARS-CoV-2 variant cannot be formally excluded [16]. As in India, the detection of Mucorales in our centre coincided with the spread of the Delta variant among ICU patients in France. Second, the widespread use of dexamethasone/tocilizumab therapy during this fourth wave, a combination that was not used or was less frequently used during the previous waves, could constitute a plausible explanation for this emergence [16]. However, since mid-September, the incidence of Mucorales detection in COVID-19 patients drastically decreased in our centre, while the Delta variant was still predominant in France and dexamethasone/tocilizumab combination therapy was still being used to treat severe COVID-19. This might be an argument against these two hypotheses. Third, environmental conditions might be responsible. The risk of acquiring IMI varies according to the level of individual exposure to fungal spores. *Aspergillus* and Mucorales are saprophytic ubiquitous moulds frequently found in hospital environments [17]. Construction and renovation activities can increase airborne fungal spore load and have been reported to be an independent risk factor for invasive fungal infections [18]. Fungal outbreaks that occur during construction and renovation are widely described in the literature and usually implicate the *Aspergillus* species, but Mucorales has also been reported [19]. In our centre, excavation and building activities occurred within the proximity of the ICU and the hypothesis of fungal dissemination via this activity cannot be excluded. The simultaneous massive increase in Mucorales and *Aspergillus* detection in August–September 2021, as well as the unusual proportion of non-*fumigati Aspergillus* species compared to the norm [20], could constitute an argument in favour of this hypothesis. Moreover, seasonal and climatic conditions may have played a role in the development of the Mucorales cluster observed in our centre. In France, the first COVID-19 wave occurred during the summer. Several studies have shown that the incidence of Mucorales infections was significantly higher during months with a mean temperature above 20 °C [21,22]. In August 2021, the mean temperature in Toulouse was 23 °C [12]. Nevertheless, no studies are available to date concerning the impact of seasons on IMI-complicated COVID infection.

Results and interpretations are limited by the retrospective design of this study. Data may be incomplete due to the absence of fungal screening by PCR for one part of the COVID-19 patients hospitalised in the ICU. Moreover, even if an association between environmental conditions (construction work, temperature) and the increased fungal detection seems likely, it remains very difficult to demonstrate a direct causal link.

In conclusion, CAM is an emerging disease with an apparently rare incidence in Europe. Nevertheless, this Mucorales cluster detected in our centre should serve as a reminder that CAM is not just an “India-specific consequence of COVID-19”. When environmental conditions (high levels of airborne spores due to construction work, local climatic conditions, such as summer temperatures) are associated with a high number of COVID-19 patients in the ICU, a CAM cluster can also develop in European countries. It is also important to remind hospital staff that the institutional COVID-driven recommendation to open the windows of tertiary rooms is not appropriate for the ICU controlled environmental zone. Early diagnosis of fungal infections is critical to start appropriate antifungal therapy and ensure patient survival [23]. Moreover, regular screening of patients at risk with sensitive methods such as PCR seems necessary, particularly when environmental conditions promote spore dissemination. The specific role of the Delta variant or COVID-19 therapy in the emergence of CAM should also be further clarified.

## Figures and Tables

**Table 1:** Characteristics of COVID-19 Patients with Mucorales-positive PCR in respiratory samples.

Patient	Period	Age	Sex	SARS-CoV-2 Variant	Underling Conditions	Dexamethasone	Tocilizumab	Serum M/R PCR	CAM Therapy	Aspergillus Associated	Death
**A**	October 2020	83	m	Undetermined	Hypertension	Yes	No	Positive	L-AmB	Yes	Yes
**B**	August 2021	72	m	Delta	No	Yes	Yes	Positive	L-AmB, PCZ	Yes	Yes
**C**	August 2021	58	f	Delta	BMI > 30, ET	Yes	Yes	Positive	L-AmB, IVZ, PCZ	Yes	No
**D**	August 2021	68	m	Delta	Hypertension, CRI	Yes	Yes	Negative	L-AmB	Yes	Yes
**E**	August 2021	39	f	Delta	BMI > 30	Yes	Yes	Negative	L-AmB, IVZ	No	No
**F**	August 2021	73	m	Undetermined	Hypertension	Yes	Yes	Negative	L-AmB, IVZ	Yes	No
**G**	August 2021	52	m	Delta	BMI > 30, hypertension	Yes	Yes	Negative	L-AmB, IVZ	Yes	No
**H**	August 2021	77	m	Delta	DM, hypertension	No	No	Negative	No	No	Yes
**I**	August 2021	70	m	Undetermined	DM, hypertension	Yes	No	Negative	L-AmB	Yes	Yes
**J**	September 2021	61	m	Delta	No	Yes	Yes	Negative	IVZ	Yes	No

ICU: intensive care unit; m: male; f: female; BMI: body mass index; CRI: chronic renal insufficiency; DM: diabetes mellitus; ET: essential thrombocytemia; M/R: *Mucor*/*Rhizopus*; CAM: COVID-19-Associated Mucormycosis; IVZ: isavuconazole; L-AmB: liposomal-amphotericin B; PCZ: posaconazole.

## Data Availability

Not applicable.
